# Experimental identification of non-classicality of noisy twin beams and other related two-mode states

**DOI:** 10.1038/s41598-018-19634-1

**Published:** 2018-01-23

**Authors:** Ievgen I. Arkhipov, Jan Peřina

**Affiliations:** RCPTM, Joint Laboratory of Optics of Palacký University and Institute of Physics of CAS, Faculty of Science, Palacký University, 17.listopadu 12, 771 46 Olomouc, Czech Republic

**Keywords:** Nonlinear optics, Quantum optics

## Abstract

Different non-classicality criteria expressed in the form of inequalities among intensity moments and elements of photon-number distributions are applied to noisy twin beams and other two-mode states obtained from a twin beam by using a beam splitter. Their performance in revealing the non-classicality is judged in comparison with the exact results provided by suitable entanglement and local non-classicality quantifiers. Whereas the non-classicality of noisy twin beams is always revealed by these criteria, not all the nonclassical states obtained at the output of the beam splitter can be identified by these experimentally easily reachable criteria.

## Introduction

Nonclassical properties of light have been in the focus of investigations in quantum nonlinear optics for a long time. Broad and deep studies of non-classicality of optical fields and especially of entanglement among their parts even resulted in establishing a new field in science — quantum information science^1,2^. Historically, discrete- and continuous-variable quantum-optical systems have been distinguished when investigating their nonclassical properties. Recently, the so-called hybrid quantum-optical systems composed of both discrete- and continuous-variable parts in the mutual interaction have been addressed. Here, we pay attention to non-classicality of a two-mode optical field that has probably been the most frequently studied system described by the continuous variables. It is well known that these fields can exhibit both entanglement between their modes^3,4^ and squeezing inside their modes^5,6^. Whereas the squeezing of the modes manifests local non-classicality of these modes, the entanglement between the modes is responsible for global non-classicality of the overall two-mode field. Moreover, the global non-classicality of the overall field is also implied by the local non-classicalities of individual modes^7^.

Different techniques have been developed to experimentally verify non-classicality of optical fields. The most elaborated, and also the most experimentally demanding, technique is homodyne tomography that relies on homodyne detection^8^ when reconstructing a quantum state^9,10^. On the other hand, the usual quadratic optical detectors recording just the field’s intensity or the fields’ intensity correlations have undergone fast development in the last ten years and their recent variants aimed at detecting weak optical fields provide photon-number resolution. This brings the opportunity to identify non-classicality under suitable conditions, even not knowing the phases of fields’ complex amplitudes. In principle, an experimental photocount histogram can be used to reconstruct the quasi-distribution of integrated intensities (or more exactly its regularized form) and reveal the non-classicality through its negative values^11,12^. Or one can just rely on the application of various non-classicality criteria usually in the form of inequalities, as it has been done, e.g., in refs^13,14^ for single-mode fields and in ref.^15^ for two-mode fields.

The importance of identification of non-classicality through the measurement with photon-number-resolving detectors is constantly growing as these detectors are becoming more efficient and so more popular. Superconducting bolometers are at present the most sophisticated detectors of this kind. They are endowed with the best quantum detection efficiencies, but at the expense of their cryogenic operation. On the other hand, the oldest fiber-based photon-number-resolving detectors with time multiplexing are relatively cheap and easy to operate^16–20^. Ultra-sensitive cameras^21,22^, especially intensified CCD (iCCD) cameras^11–13,23,24^, can also be applied as massively spatially-multiplexed photon-number-resolving detectors. Due to large numbers of pixels on their photocathodes, these cameras are suitable also for characterizing mesoscopic optical fields^25^. Hybrid detectors^26^ and also silicon multi-pixel detection arrays^27^ also belong to prospective photon-number-resolving detectors at present.

When applying various non-classicality criteria an important question about their performance arises. Namely, how many nonclassical states can be revealed by these criteria. Also mutual comparison of different criteria has important consequences for their practical use. Many non-classicality inequalities applied to specific kinds of both single mode and two-mode optical fields have been mutually compared in refs^13–15^ using the experimental data. The applied non-classicality inequalities have shown good experimental performance in the cases of sub-Poissonian states and practically noiseless twin beams. This poses the question about their power in the cases of other nonclassical states that can be relatively easily reached in the laboratory. Here, we theoretically address two such kinds of states: noisy twin beams and two-mode states derived from the noisy twin beams using, in general, an unbalanced beam splitter. Especially the second kind of states is interesting as it allows, via the bunching effect of photon pairs at a beam splitter, to obtain squeezed single-mode states^3,4,7,28^, that exhibit local non-classicality. To simulate the experimental identification of non-classicality we apply the non-classicality criteria written in either the normally-ordered intensity moments or the elements of photon-number (photocount) distributions that have been developed and summarized in ref.^15^. To theoretically judge local non-classicality and entanglement of the analyzed states we invoke the local non-classicality and entanglement quantifiers derived in refs^29,30^, that are especially suitable for two-mode Gaussian states and their transformations at a beam splitter. We show that whereas the applied global non-classicality criteria (GNCCa) allow us to recognize all entangled noisy twin beams, not all two-mode nonclassical states occurring beyond the beam splitter can be identified with the used GNCCa and local non-classicality criteria (LNCCa). Our analysis also shows that the criteria based on the elements of photon-number distributions exhibit better performance compared to those written in intensity moments. We also identify the most powerful criteria.

The paper is organized as follows. Section *Two-mode optical fields and their properties* brings the description of nonclassical properties of optical fields. The analyzed non-classicality criteria are mentioned in section *Non-classicality criteria*. The states originating in the noisy twin beams propagating through an unbalanced beam splitter are discussed in section *Twin beam and its transformation on a beam splitter*. Section *Identification of non-classicality of twin beams* is devoted to the performance of the used non-classicality criteria in revealing the entanglement of noisy twin beams. Similarly, different non-classicality criteria are applied in section *Identification of non-classicality of two-mode states beyond a beam splitter* to reveal non-classicality of the addressed states. In section *Conclusions* the main findings are summarized.

### Two-mode optical fields and their properties

Any two-mode state characterized by its density matrix $\hat{\rho }$ can be expressed in the Glauber-Sudarshan (diagonal) representation based on coherent states |α_1_〉 and |α_2_〉 defined in modes 1 and 2, respectively:

1 $$\hat{\rho }=\frac{1}{{\pi }^{2}}\int {d}^{2}{\alpha }_{1}\int {d}^{2}{\alpha }_{2}{\mathscr{P}}({\alpha }_{1},{\alpha }_{2})|{\alpha }_{1}\rangle |{\alpha }_{2}\rangle \langle {\alpha }_{2}|\langle {\alpha }_{1}|\cdot $$

In Eq. (1), ${\mathscr{P}}({\alpha }_{1},{\alpha }_{2})$ stands for the Glauber-Sudarshan quasi-distribution^31,32^. The quasi-distribution *P* uniquely identifies nonclassical states. If it attains the form of a regular distribution function with non-negative values (or has the form of a sum of the Dirac *δ*-functions) it describes a classical state. However, if it becomes negative or even more singular than the Dirac *δ*-function, it corresponds to a nonclassical state.

If the information about the phase of an optical field is not known, we can restrict our attention to quasi-distribution *P*(*W*_1_, *W*_2_) of integrated intensities *W*_1_ and *W*_2_ (*W*_*j*_ = |α_*j*_|^2^, *j* = 1, 2)^6^, instead of using the Glauber-Sudarshan quasi-distribution ${\mathscr{P}}({\alpha }_{1},{\alpha }_{2})$. Moments $\langle {W}_{1}^{{k}_{1}}{W}_{2}^{{k}_{2}}\rangle$, *k*_1_, *k*_2_ = 0, 1, …, of the integrated intensities *W*_1_ and *W*_2_ (farther only intensities) are then easily determined by averaging with the intensity quasi-distribution *P*(*W*_1_, *W*_2_):

2 $$\langle {W}_{1}^{{k}_{1}}{W}_{2}^{{k}_{2}}\rangle ={\int }_{0}^{\infty }d{W}_{1}{\int }_{0}^{\infty }d{W}_{2}P({W}_{1},{W}_{2}){W}_{1}^{{k}_{1}}{W}_{2}^{{k}_{2}}\cdot $$

We note that the intensity moments 〈*W*^k^〉 are just the normally ordered moments ${\hat{a}}^{\dagger k}{\hat{a}}^{k}$ of the photon-number operator as $\langle {W}^{k}\rangle \equiv \langle {\hat{a}}^{\dagger k}{\hat{a}}^{k}\rangle$ and ${\hat{a}}^{\dagger }$ ($\hat{a}$) stands for the creation (annihilation) operator.

According to the Mandel photodetection formula^6^, photon-number distribution *p*(*n*_1_, *n*_2_) for a field with intensity quasi-distribution *P*(*W*_1_, *W*_2_) is obtained as follows:

3 $$p({n}_{1},{n}_{2})=\frac{1}{{n}_{1}!{n}_{2}!}{\int }_{0}^{\infty }d{W}_{1}{\int }_{0}^{\infty }d{W}_{2}P({W}_{1},{W}_{2}){W}_{1}^{{n}_{1}}{W}_{2}^{{n}_{2}}\exp [-({W}_{1}+{W}_{2})]\cdot $$

Both the intensity moments given in Eq. (2) and the elements of photon-number distribution *p* written in Eq. (3) can conveniently be derived from the normal generating function ${{\rm{G}}}_{{\mathscr{N}}}$ defined as:

4 $${G}_{{\mathscr{N}}}({\lambda }_{1},{\lambda }_{2})={\int }_{0}^{\infty }d{W}_{1}{\int }_{0}^{\infty }d{W}_{2}P({W}_{1},{W}_{2})\exp (-{\lambda }_{1}{W}_{1}-{\lambda }_{2}{W}_{2})\cdot $$

Whereas the intensity moments $\langle {W}_{1}^{{k}_{1}}{W}_{2}^{{k}_{2}}\rangle$ are obtained along the formula

5 $$\langle {W}_{1}^{{k}_{1}}{W}_{2}^{{k}_{2}}\rangle ={(-1)}^{{k}_{1}+{k}_{2}}{\frac{{\partial }^{{k}_{1}+{k}_{2}}{G}_{{\mathscr{N}}}({\lambda }_{1},{\lambda }_{2})}{\partial {\lambda }_{1}^{{k}_{1}}\partial {\lambda }_{2}^{{k}_{2}}}|}_{{\lambda }_{1}={\lambda }_{2}=0},$$

the elements *p*(*n*_1_, *n*_2_) of photon-number distribution are reached as follows:

6 $$p({n}_{1},{n}_{2})=\frac{{(-1)}^{{n}_{1}+{n}_{2}}}{{n}_{1}!{n}_{2}!}{\frac{{\partial }^{{n}_{1}+{n}_{2}}{G}_{{\mathscr{N}}}({\lambda }_{1},{\lambda }_{2})}{\partial {\lambda }_{1}^{{n}_{1}}\partial {\lambda }_{2}^{{n}_{2}}}|}_{{\lambda }_{1}={\lambda }_{2}=1}.$$

### Non-classicality criteria

We describe non-classicality criteria that have been derived in ref.^15^ and have shown the best performance. The violation of the classical inequality

7 $$\langle {W}_{1}^{{k}_{1}}{W}_{2}^{{k}_{2}}{({W}_{1}-{W}_{2})}^{2}\rangle  > 0$$

gives us the following GNCCa for *k*_1_, *k*_2_ ≥ 0:

8 $${E}_{{k}_{1},{k}_{2}}^{W}\equiv \langle {W}_{1}^{{k}_{1}+2}{W}_{2}^{{k}_{2}}\rangle +\langle {W}_{1}^{{k}_{1}}{W}_{2}^{{k}_{2}+2}\rangle -2\langle {W}_{1}^{{k}_{1}+1}{W}_{2}^{{k}_{2}+1}\rangle  < 0.$$

Following the correspondence between the GNCCa based on intensity moments and the GNCCa containing the elements of photon-number distribution discussed in ref.^15^. We arrive at the following GNCCa:

9 $${E}_{{k}_{1},{k}_{2}}^{p}\equiv \tilde{p}({k}_{1}+\mathrm{2,}\,{k}_{2})+\tilde{p}({k}_{1},\,{k}_{2}+2)-2\tilde{p}({k}_{1}+\mathrm{1,}\,{k}_{2}+1) < 0$$

using the modified elements $\tilde{p}({n}_{1},{n}_{2})$ of photon-number distribution,

10 $$\tilde{p}({n}_{1},{n}_{2})=\frac{{n}_{1}!{n}_{2}!p({n}_{1},{n}_{2})}{p(\mathrm{0,}\,0)}\mathrm{.}$$

Also the GNCCa *M*^*W*^ and *M*^*P*^ defined along the relations

11 $$\begin{array}{l}{M}^{W}\equiv \langle {W}_{1}^{2}\rangle \langle {W}_{2}^{2}\rangle -{\langle {W}_{1}{W}_{2}\rangle }^{2} < \mathrm{0,}\\ {M}^{p}\equiv \tilde{p}(\mathrm{2,}\,0)\tilde{p}(\mathrm{0,}\,2)-\tilde{p}{(\mathrm{1,}1)}^{2} < 0\end{array}$$

have been found powerful in ref.^15^ when revealing non-classicality. We note that they originate in the matrix approach^33–35^ that is based upon non-negativity of classical quadratic forms.

The most powerful single-mode LNCCa have been derived in ref.^36^ using the majorization theory. They have been tested on the experimental sub-Poissonian fields in ref.^13^. They attain the following form for mode *j*, *j* = 1, 2:

12 $$\begin{array}{c}{R}_{k,l}^{{W}_{j}}\equiv \langle {W}_{j}^{k+1}\rangle \langle {W}_{j}^{l-1}\rangle -\langle {W}_{j}^{k}\rangle \langle {W}_{j}^{l}\rangle  < \mathrm{0,}\\ {R}_{k,l}^{{p}_{j}}\equiv {\tilde{p}}_{j}(k+1){\tilde{p}}_{j}(l-1)-{\tilde{p}}_{j}(k){\tilde{p}}_{j}(l) < 0.\end{array}$$

The modified elements $${\tilde{p}}_{j}(n)$$ of marginal photon-number distribution *p*_*j*_(*n*) of mode *j* are given as$${\tilde{p}}_{j}(n)=$$$$n!{p}_{j}(n)/{p}_{j}(0)$$.

### Twin beam and its transformation on a beam splitter

A twin beam is generated in the process of spontaneous parametric down-conversion that generates photon pairs at the expense of annihilated pump photons^5^. Twin beams in general contain more photon pairs and they can also contain an additional noise in the form of individual photons^6^. Such general noisy twin beams belong to two-mode Gaussian optical fields that can be conveniently described by the normal quantum characteristic function ${C}_{{\mathscr{N}}}$ defined as^6^

13 $$\begin{array}{rcl}{C}_{{\mathscr{N}}}({\beta }_{1},{\beta }_{2}) & = & \langle \exp ({\beta }_{1}{\hat{a}}_{1}^{\dagger }+{\beta }_{2}{\hat{a}}_{2}^{\dagger })\exp (-{\beta }_{1}^{\ast }{\hat{a}}_{1}-{\beta }_{2}^{\ast }{\hat{a}}_{2})\rangle \\  & = & \int {d}^{2}{\alpha }_{1}\int {d}^{2}{\alpha }_{2}\prod _{j=1}^{2}\exp ({\beta }_{j}{\alpha }_{j}^{\ast }-{\beta }_{j}^{\ast }{\alpha }_{j}){\mathscr{P}}({\alpha }_{1},{\alpha }_{2})\end{array}$$

using the Glauber-Sudarshan quasi-distribution ${\mathscr{P}}$.

Both the noisy twin beams and the states arising beyond a beam splitter with an impinging twin beam belong to two-mode Gaussian states with the following form of the normal characteristic function ${C}_{{\mathscr{N}}}$:

14 $${C}_{{\mathscr{N}}}({\beta }_{1},{\beta }_{2})=\exp [-{B}_{1}{|{\beta }_{1}|}^{2}-{B}_{2}{|{\beta }_{2}|}^{2}+(\frac{{C}_{1}}{2}{\beta }_{1}^{\ast 2}+\frac{{C}_{2}}{2}{\beta }_{2}^{\ast 2}+{D}_{12}{\beta }_{1}^{\ast }{\beta }_{2}^{\ast }+{\bar{D}}_{12}{\beta }_{1}{\beta }_{2}^{\ast }+{\rm{c}}\mathrm{.}{\rm{c}}\mathrm{.})];$$

symbol c.c. replaces the complex-conjugated terms. The coefficients *B*_*j*_*C*_*j*_, *j* = 1,2, *D*_12_, and ${\overline{D}}_{12}$ introduced in Eq. (14) are defined as follows:

15 $$\begin{array}{cc}{B}_{j}=\langle {\rm{\Delta }}{\hat{a}}_{j}^{\dagger }{\rm{\Delta }}{\hat{a}}_{j}\rangle , & {C}_{j}=\langle {({\rm{\Delta }}{\hat{a}}_{j})}^{2}\rangle ,\\ {D}_{12}=\langle {\rm{\Delta }}{\hat{a}}_{1}{\rm{\Delta }}{\hat{a}}_{2}\rangle , & {\overline{D}}_{12}=-\,\langle {\rm{\Delta }}{\hat{a}}_{1}^{\dagger }{\rm{\Delta }}{\hat{a}}_{2}\rangle ,\end{array}$$

and $$\triangle {\hat{\alpha }}_{j}\equiv {\hat{\alpha }}_{j}-\langle {\hat{\alpha }}_{j}\rangle $$.

The normal characteristic function ${C}_{{\mathscr{N}}}$ given in Eq. (14) can conveniently be rewritten into the form ${C}_{{\mathscr{N}}}({\boldsymbol{\beta }})=\exp ({{\boldsymbol{\beta }}}^{\dagger }{{\bf{A}}}_{\pmb{\mathscr{N}}}{\boldsymbol{\beta }}/2)$ using the covariance matrix ${{\bf{A}}}_{{\mathscr{N}}}$ related to normal ordering of the field operators^6,37^,

16 $${{\bf{A}}}_{{\mathscr{N}}}=[\begin{array}{cccc}-{B}_{1} & {C}_{1} & {\overline{D}}_{12}^{\ast } & {D}_{12}\\ {C}_{1}^{\ast } & -{B}_{1} & {D}_{12}^{\ast } & {\overline{D}}_{12}\\ {\overline{D}}_{12} & {D}_{12} & -{B}_{2} & {C}_{2}\\ {D}_{12}^{\ast } & {\overline{D}}_{12}^{\ast } & {C}_{2}^{\ast } & -{B}_{2}\end{array}],$$

and the column vector *β* is given as ${\boldsymbol{\beta }}\equiv {({\beta }_{1},{\beta }_{1}^{\ast },{\beta }_{2},{\beta }_{2}^{\ast })}^{T}$.

The normally-ordered generating function ${{G}}_{{\mathscr{N}}}$ from Eq. (4) is then obtained along the formula^38^:

17 $${G}_{{\mathscr{N}}}({\lambda }_{1},{\lambda }_{2})=\frac{1}{{\pi }^{2}{\lambda }_{1}{\lambda }_{2}}\int {d}^{2}{\beta }_{1}\int {d}^{2}{\beta }_{2}\exp (-\frac{{|{\beta }_{1}|}^{2}}{{\lambda }_{1}}-\frac{{|{\beta }_{2}|}^{2}}{{\lambda }_{2}}){C}_{{\mathscr{N}}}({\beta }_{1},{\beta }_{2})\mathrm{.}$$

Considering the form of the characteristic function ${C}_{{\mathscr{N}}}$ written in Eq. (14), we arrive at the generating function *G*_*N*_ for the considered states:

18 $${G}_{{\mathscr{N}}}({\lambda }_{1},{\lambda }_{2})=\frac{1}{{[{{\boldsymbol{\lambda }}}^{T}{\bf{K}}{\boldsymbol{\lambda }}]}^{1/2}}$$

and **λ** ≡ (1, λ_1_, λ_2_, λ_1_λ_2_)^*T*^ The matrix **K** occurring in Eq. (18) is obtained as

19 $${\bf{K}}=[\begin{array}{cccc}1 & {K}_{12} & {K}_{13} & {K}_{14}\\ 0 & {K}_{22} & 0 & {K}_{24}\\ 0 & 0 & {K}_{33} & {K}_{34}\\ 0 & 0 & 0 & {K}_{44}\end{array}],$$

$$\begin{array}{rcl}{K}_{12} & = & 2{B}_{1},\\ {K}_{13} & = & 2{B}_{2},\\ {K}_{14} & = & 4{B}_{1}{B}_{2}-\mathrm{2|}{D}_{12}{|}^{2}-\mathrm{2|}{\bar{D}}_{12}{|}^{2},\\ {K}_{22} & = & {B}_{1}^{2}-|{C}_{1}{|}^{2},\\ {K}_{24} & = & 2{B}_{1}^{2}{B}_{2}-2{B}_{1}(|{D}_{12}{|}^{2}+|{\bar{D}}_{12}{|}^{2})\\  &  & -\,2{B}_{2}|{C}_{1}{|}^{2}-4{\rm{R}}e[{C}_{1}{\bar{D}}_{12}{D}_{12}^{\ast }],\\ {K}_{33} & = & {B}_{2}^{2}-|{C}_{2}{|}^{2},\\ {K}_{34} & = & 2{B}_{1}{B}_{2}^{2}-2{B}_{2}(|{D}_{12}{|}^{2}+|{\bar{D}}_{12}{|}^{2})\\  &  & -\,2{B}_{1}|{C}_{2}{|}^{2}-4{\rm{R}}e[{C}_{2}{\bar{D}}_{12}^{\ast }{D}_{12}^{\ast }],\\ {K}_{44} & = & {B}_{1}^{2}{B}_{2}^{2}+|{D}_{12}{|}^{2}+|{\bar{D}}_{12}{|}^{2}+|{C}_{1}{|}^{2}|{C}_{2}{|}^{2}\\  &  & -\,{B}_{1}^{2}|{C}_{2}{|}^{2}-{B}_{2}^{2}|{C}_{1}{|}^{2}\\  &  & -\,2{B}_{1}{B}_{2}|{D}_{12}{|}^{2}-\mathrm{2|}{D}_{12}{|}^{2}|{\bar{D}}_{12}{|}^{2}\\  &  & -\,4{B}_{1}{\rm{R}}e[{C}_{2}{\bar{D}}_{12}^{\ast }{D}_{12}^{\ast }]-4{B}_{2}{\rm{R}}e[{C}_{1}{\bar{D}}_{12}{D}_{12}^{\ast }]\\  &  & -\,2{\rm{R}}e[{C}_{1}{C}_{2}{D}_{12}^{\mathrm{\ast 2}}]-2{\rm{R}}e[{C}_{1}{C}_{2}^{\ast }{\bar{D}}_{12}^{2}]\mathrm{.}\end{array}$$

The considered noisy twin beams are characterized by the following parameters^6,39^

20 $$\begin{array}{c}{B}_{1}={B}_{{\rm{p}}}+{B}_{{\rm{s}}},\,{B}_{2}={B}_{{\rm{p}}}+{B}_{{\rm{i}}},\,{D}_{12}=i\sqrt{{B}_{{\rm{p}}}({B}_{{\rm{p}}}+\mathrm{1)}},\\ {C}_{1}={C}_{2}={\bar{D}}_{12}=0\end{array}$$

where *B*_P_ is the mean photon-pair number and *B*_S_ (*B*_i_) stands for the mean signal (idler) noise photon number.

The transformation of a twin beam through the beam splitter can be treated at the level of its covariance matrix ${{\bf{A}}}_{{\mathscr{N}}}$. The covariance matrix ${{\bf{A}}}_{{\mathscr{N}}}^{{\rm{out}}}$ appropriate for the state at the output of a beam splitter with transmissivity *T* is found as ${{\bf{A}}}_{{\mathscr{N}}}^{{\rm{out}}}={{\bf{U}}}^{\dagger }{{\bf{A}}}_{{\mathscr{N}}}^{{\rm{in}}}{\bf{U}}$, where the covariance matrix ${{\bf{A}}}_{{\mathscr{N}}}^{{\rm{in}}}$ characterizes the impinging twin beam and symbol **U** stands for the following unitary matrix:

21 $${\bf{U}}=(\begin{array}{cccc}\sqrt{T} & 0 & -\sqrt{R}\exp (i\varphi ) & 0\\ 0 & \sqrt{T} & 0 & -\sqrt{R}\exp (\,-\,i\varphi )\\ \sqrt{R}\exp (\,-\,i\varphi ) & 0 & \sqrt{T} & 0\\ 0 & \sqrt{R}\exp (i\varphi ) & 0 & \sqrt{T}\end{array}),$$

*R* = 1 − *T*. The phase *ϕ* occurring in Eq. (21) can be set to zero without the loss of generality. The application of the beam-splitter transformation (21) to an input noisy twin beam with parameters given in Eq. (20) leaves us with the following two-mode Gaussian state:

22 $$\begin{array}{c}{B}_{1}^{{\rm{o}}ut}=\,-\,T{B}_{{\rm{s}}}-{B}_{{\rm{p}}}-R{B}_{{\rm{i}}},\\ {B}_{2}^{{\rm{o}}ut}=\,-\,T{B}_{{\rm{i}}}-{B}_{{\rm{p}}}-R{B}_{{\rm{s}}},\\ {C}_{1}^{{\rm{o}}ut}=\,-\,{C}_{2}^{{\rm{o}}ut}\mathrm{=2}i\sqrt{TR}\sqrt{{B}_{{\rm{p}}}({B}_{{\rm{p}}}+\mathrm{1)}},\\ {D}_{12}^{{\rm{o}}ut}=\,i\mathrm{(2}T-\mathrm{1)}\sqrt{{B}_{{\rm{p}}}({B}_{{\rm{p}}}+\mathrm{1)}},\\ {\bar{D}}_{12}^{{\rm{o}}ut}=\,\sqrt{TR}({B}_{{\rm{s}}}-{B}_{{\rm{i}}}\mathrm{).}\end{array}$$

Alternatively, we may derive explicit formulas for photon-number distributions of both the impinging noisy twin beam and the state at the output of the beam splitter. The photon-number distribution *p*(*n*_1_, *n*_2_) of a noisy twin beam has been found in ref.^39^:

23 $$p({n}_{1},{n}_{2})=\frac{1}{\tilde{K}}\sum _{m\mathrm{=0}}^{{\rm{\min }}({n}_{1},{n}_{2})}(\begin{array}{c}{n}_{1}\\ m\end{array})(\begin{array}{c}{n}_{2}\\ m\end{array}){(1-\frac{{\tilde{B}}_{1}}{\tilde{K}})}^{{n}_{2}-m}{(1-\frac{{\tilde{B}}_{2}}{\tilde{K}})}^{{n}_{1}-m}{(\frac{|{D}_{12}|}{\tilde{K}})}^{2m},$$

where ${\tilde{B}}_{j}={B}_{j}+1$ for *j* = 1, 2 and $\tilde{K}={\tilde{B}}_{1}{\tilde{B}}_{2}-|{D}_{12}{|}^{2}$. On the other hand, the photon-number distribution *p*^*out*^(*n*_1_, *n*_2_) of the state at the beam-splitter output is determined along the formula^7^:

24 $${p}^{{\rm{o}}ut}({n^{\prime} }_{1},{n^{\prime} }_{2})=\sum _{{n}_{1}\mathrm{=0}}^{\infty }\sum _{{n}_{2}\mathrm{=0}}^{\infty }{B}_{{n}_{1},{n}_{2}}^{{n^{\prime} }_{1},{n^{\prime} }_{2}}p({n}_{1},{n}_{2}\mathrm{).}$$

The coefficients ${B}_{{n}_{1},{n}_{2}}^{{n^{\prime} }_{1},{n^{\prime} }_{2}}$ in Eq. (24) are defined as

25 $${B}_{{n}_{1},{n}_{2}}^{{n^{\prime} }_{1},{n^{\prime} }_{2}}=\sum _{{k}_{1}\mathrm{=0}}^{{n}_{1}}\sum _{{k}_{2}\mathrm{=0}}^{{n}_{2}}{(-1)}^{{n}_{1}-{k}_{1}}{\sqrt{R}}^{{n}_{1}+{n}_{2}-{k}_{1}-{k}_{2}}{\sqrt{T}}^{{k}_{1}+{k}_{2}}\frac{\sqrt{{n}_{1}!{n}_{2}!{n^{\prime} }_{1}!{n^{\prime} }_{2}!}}{{k}_{1}!({n}_{1}-{k}_{1})!{k}_{2}!({n}_{2}-{k}_{2})!}{\delta }_{{n^{\prime} }_{1},{n}_{2}+{k}_{1}-{k}_{2}}{\delta }_{{n^{\prime} }_{2},{n}_{1}-{k}_{1}+{k}_{2}}$$

and *δ* means the Kronecker symbol.

The local non-classicality quantifiers ${I}_{{\rm{n}}cl}^{(j)}$, *j* = 1, 2 and entanglement quantifier *I*_ent_ introduced in ref.^30^ have been found suitable as theoretical characteristics for the analyzed two-mode Gaussian states. The reason is that these quantifiers together form the global non-classicality invariant *I*_ncl_,

26 $${I}_{{\rm{ncl}}}={I}_{{\rm{ncl}}}^{\mathrm{(1)}}+{I}_{{\rm{ncl}}}^{\mathrm{(1)}}+2{I}_{{\rm{ent}}},$$

that does not change when any photon-number preserving unitary transformation is applied. According to refs.^29,30^ the local non-classicality quantifier ${I}_{{\rm{ncl}}}^{(j)}$ for mode *j* is given as:

27 $${I}_{{\rm{ncl}}}^{(j)}=-\,{B}_{j}^{2}+|{C}_{j}{|}^{2}\mathrm{.}$$

On the other hand, both three local and one global invariants of the symmetrically-ordered covariance matrix *A*_*S*_ are needed to determine the entanglement quantifier *I*_ent_. Details can be found in ref.^29^.

### Identification of non-classicality of twin beams

We first consider the simplest case of a noiseless twin beam whose only parameter is the mean photon-pair number *B*_P_. Its entanglement, which is responsible for its non-classicality, has been theoretically analyzed in ref.^39^ where negativity *N*, which is an entanglement measure^40^, has been derived as $N=\sqrt{{B}_{{\rm{p}}}({B}_{{\rm{p}}}+\mathrm{1)}}+{B}_{{\rm{p}}}$. Thus, the entanglement of a noiseless twin beam increases with the photon-pair number *B*_P_. In our analysis, we consider the first five GNCCa ${E}_{{k}_{1},{k}_{2}}^{W}$ and ${E}_{{k}_{1},{k}_{2}}^{p}$ that contain the intensity moments up to the sixth order and the elements of photon-number distribution for up to six photons. We note that the consideration of lower-order intensity moments is natural as the experimental error increases with the increasing order of intensity moments.

The GNCCa *E*^*W*^ and *M*^*W*^ given in Eqs. (8) and (11), respectively, and using intensity moments attain in the case of a noiseless twin beam the forms:

28 $$\begin{array}{rcl}{E}_{\mathrm{0,0}}^{W} & = & -\,2{B}_{{\rm{p}}},\\ {E}_{\mathrm{1,1}}^{W} & = & -\,12{B}_{{\rm{p}}}^{3}-8{B}_{{\rm{p}}}^{2},\\ {E}_{\mathrm{2,2}}^{W} & = & -\,240{B}_{{\rm{p}}}^{5}-288{B}_{{\rm{p}}}^{4}-72{B}_{{\rm{p}}}^{3},\\ {E}_{\mathrm{0,1}}^{W} & = & -\,4{B}_{{\rm{p}}}^{2},\\ {E}_{\mathrm{0,2}}^{W} & = & -\,12{B}_{{\rm{p}}}^{3}+4{B}_{{\rm{p}}}^{2},\end{array}$$

29 $${M}^{W}=-\,4{B}_{{\rm{p}}}^{3}-{B}_{{\rm{p}}}^{2}\mathrm{.}$$

On the other hand, their counterparts *E*^*P*^ and *M*^*P*^ involving the elements of photon-number distribution and written in Eqs. (9) and (11), respectively, are obtained as:

30 $$\begin{array}{rcl}{E}_{\mathrm{0,0}}^{p} & = & -\,2{B}_{{\rm{p}}}/({B}_{{\rm{p}}}+\mathrm{1),}\\ {E}_{\mathrm{1,1}}^{p} & = & -\,8{B}_{{\rm{p}}}^{2}/({B}_{{\rm{p}}}+{\mathrm{1)}}^{2},\\ {E}_{\mathrm{2,2}}^{p} & = & -\,72{B}_{{\rm{p}}}^{3}/({B}_{{\rm{p}}}+{\mathrm{1)}}^{3},\\ {E}_{\mathrm{0,1}}^{p} & = & \mathrm{0,}\\ {E}_{\mathrm{0,2}}^{p} & = & 4{B}_{{\rm{p}}}^{2}/({B}_{{\rm{p}}}+{\mathrm{1)}}^{2},\end{array}$$

31 $${M}_{p}=-\,\frac{{B}_{{\rm{p}}}^{2}}{{({B}_{{\rm{p}}}+\mathrm{1)}}^{2}}\mathrm{.}$$

**Figure 1:**
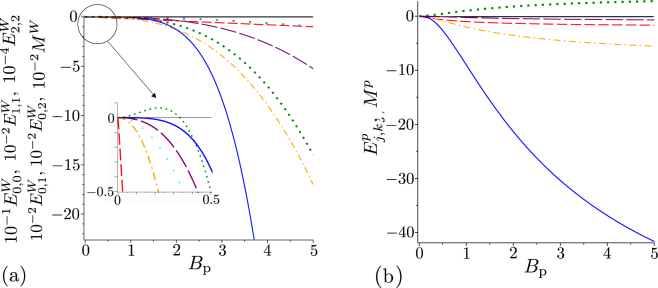
(**a**) GNCCa ${E}_{\mathrm{0,0}}^{W}$ (red dashed curve), ${E}_{\mathrm{1,1}}^{W}$ (orange dash-dotted curve), ${E}_{\mathrm{2,2}}^{W}$ (blue solid curve), ${E}_{\mathrm{0,1}}^{W}$ (cyan long dotted curve), ${E}_{\mathrm{0,2}}^{W}$ (green dotted curve), and *M*^*W*^ (purple long dashed curve) and (**b**) GNCCa ${E}_{\mathrm{0,0}}^{p}$ (red dashed curve), ${E}_{\mathrm{1,1}}^{p}$ (orange dash-dotted curve), ${E}_{\mathrm{2,2}}^{p}$ (blue solid line), ${E}_{\mathrm{0,2}}^{p}$ (green dotted curve), and *M*^*P*^ (purple long dashed curve) as they depend on photon-pair number *B*_P_ for noiseless twin beams.

Mutual comparison of the formulas for GNCCa written in Eqs (28)—(31) reveals qualitatively different behavior of these GNCCa for greater photon-pair numbers *B*_P_ (see Fig. 1). Whereas the GNCCa based on intensity moments tend to go to minus infinity, the GNCCa using the elements of photon-number distributions reach finite values for *B*_P_→∞.

The GNCCa ${E}_{k,k}^{W}$, *k* = 0, 1, …, ${E}_{\mathrm{0,1}}^{W}$, and *M*^*W*^ as well as the GNCCa ${E}_{k,k}^{p}$, *k* = 0, 1, …, and *M*^*P*^ are entanglement monotones, since their absolute values increase with the increasing photon-pair number *B*_P_. As such, all of them (with the inverted sign) can be chosen as a suitable non-classicality identifier for any noiseless twin beam. We note that this ability to reveal the non-classicality is preserved for non-ideal detection with a finite detection efficiency.

On the other hand, the GNCC ${E}_{\mathrm{0,2}}^{W}$ can be successfully applied only for ${B}_{{\rm{p}}}\in \mathrm{(1/3,}\,\infty )$ and the GNCC ${E}_{\mathrm{0,2}}^{p}$ even attains positive values for any value of *B*_P_ A more general analysis has shown that the GNCCa ${E}_{{k}_{1},{k}_{2}}^{W}$ for |*k*_1_−*k*_2_| > 1 reveal non-classicality only for more intense twin beams and the GNCCa ${E}_{{k}_{1},{k}_{2}}^{p}$ for *k*_1_ ≠ *k*_2_ cannot indicate non-classicality at all.

**Figure 2:**
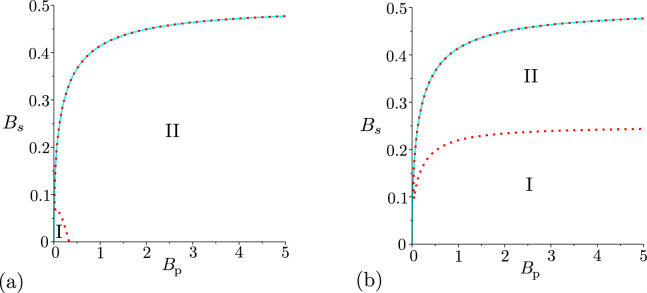
Non-classicality phase diagrams for noisy twin beams with balanced noise: (**a**) GNCCa ${E}_{\mathrm{0,0}}^{W}$, ${E}_{\mathrm{1,1}}^{W}$, ${E}_{\mathrm{2,2}}^{W}$ (coinciding cyan solid curves), and ${E}_{\mathrm{0,2}}^{W}$ (red dotted curve) and (**b**) GNCCa ${E}_{\mathrm{0,0}}^{p}$, ${E}_{\mathrm{1,1}}^{p}$, ${E}_{\mathrm{2,2}}^{p}$ (coinciding cyan solid curves), and ${E}_{\mathrm{0,2}}^{p}$ (red dashed curve) in space (*B*_S_,*B*_P_). For comparison, phase diagram of theoretical entanglement quantifier *I*ent is plotted in (**a**) and (**b**) by cyan solid curve. The GNCCa ${E}_{\mathrm{0,2}}^{W}$ and ${E}_{\mathrm{0,2}}^{p}$ express non-classicality only in region II, the remaining GNCCa are negative in regions I and II.

Now we pay attention to noisy twin beams, first considering the beams with balanced noise for which the signal and idler mean noise photon numbers equal (*B*_s_ = *B*_i_). The GNCCa ${E}_{k,k}^{W}$, *k* = 0, 1, …, and *M*^*W*^ and the GNCCa ${E}_{k,k}^{p}$, *k* = 0, 1, …, and *M*^*P*^ still fully identify non-classicality of such noisy twin beams, that is, however, observed only for twin beams with smaller amount of the noise (see Fig. 2). As it has been found in ref.^39^, only the twin beams with ${B}_{{\rm{s}}}={B}_{{\rm{i}}} < \sqrt{{B}_{{\rm{p}}}({B}_{{\rm{p}}}+\mathrm{1)}}-{B}_{{\rm{p}}}$ are nonclassical.

Interestingly, the GNCCa ${E}_{\mathrm{0,2}}^{W}$ and ${E}_{\mathrm{0,2}}^{p}$ indicate non-classicality of noisy twin beams with photon-pair numbers *B*_P_ for which they failed in the case of noiseless twin beams. This occurs for the GNCC ${E}_{\mathrm{0,2}}^{W}$ in region *B*_P_ ∈ (0,1/3) for the noisy twin beams with ${B}_{{\rm{s}}}={B}_{{\rm{i}}}\in ([\sqrt{{B}_{{\rm{p}}}({B}_{{\rm{p}}}+\mathrm{1)}}-{B}_{{\rm{p}}}\mathrm{]/2,}\sqrt{{B}_{{\rm{p}}}({B}_{{\rm{p}}}+\mathrm{1)}}-{B}_{{\rm{p}}})$ [region II in Fig. 2(a)]. Similarly, the GNCC ${E}_{\mathrm{0,2}}^{p}$, that is nonnegative for noiseless twin beams, is negative for the noisy twin beams with ${B}_{{\rm{s}}}={B}_{{\rm{i}}}\in ([\sqrt{4{B}_{{\rm{p}}}({B}_{{\rm{p}}}+\mathrm{1)}+2\sqrt{{B}_{{\rm{p}}}({B}_{{\rm{p}}}+\mathrm{1)}}+1}-2{B}_{{\rm{p}}}-\mathrm{1]/2,}\sqrt{{B}_{{\rm{p}}}({B}_{{\rm{p}}}+\mathrm{1)}}-{B}_{{\rm{p}}})$ [region II in Fig. 2(b)].

**Figure 3:**
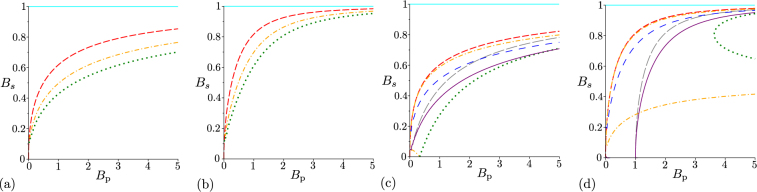
Non-classicality phase diagrams for noisy twin beams with unbalanced noise (*B*_i_ = 0): (**a**) GNCCa ${E}_{\mathrm{0,0}}^{W}$ (red dashed curve), ${E}_{\mathrm{1,1}}^{W}$ (orange dash-dotted curves), ${E}_{\mathrm{2,2}}^{W}$ (green dotted curve), and ${M}^{W}$ (cyan solid curve), (**b**) GNCCa ${E}_{\mathrm{0,0}}^{p}$ (red dashed curve), ${E}_{\mathrm{1,1}}^{p}$ (orange dash-dotted curve), ${E}_{\mathrm{2,2}}^{p}$ (green dotted curve), and *M*^*P*^ (cyan solid curve), (**c**) ${E}_{\mathrm{1,0}}^{W}$ (red dashed curve), ${E}_{\mathrm{0,1}}^{W}$ (grey long dashed curve), ${E}_{\mathrm{2,0}}^{W}$ (orange dash-dotted curves), ${E}_{\mathrm{0,2}}^{W}$ (green dotted curve), ${E}_{\mathrm{2,1}}^{W}$ (blue space dashed curve), and ${E}_{\mathrm{1,2}}^{W}$ (purple solid curve) and (**d**) GNCCa ${E}_{\mathrm{1,0}}^{p}$ (red dashed curve), ${E}_{\mathrm{0,1}}^{p}$ (grey long dashed curve), ${E}_{\mathrm{2,0}}^{p}$ (orange dash-dotted curves), ${E}_{\mathrm{0,2}}^{p}$ (green dotted curves), ${E}_{\mathrm{2,1}}^{p}$ (blue space dashed curve), and ${E}_{\mathrm{1,2}}^{p}$ (purple solid curve) in space (*B*_s_,*B*_p_). For comparison, phase diagram of entanglement quantifier *I*_ent_ is plotted by cyan solid curve in (**a**–**d**). For GNCCa ${E}_{\mathrm{2,0}}^{W}$ plotted in (**c**) and ${E}_{\mathrm{2,0}}^{p}$ [${E}_{\mathrm{0,2}}^{p}$] drawn in (**d**), the non-classicality region lies between the lower and upper orange dash-dotted [green dotted] curves. For the other GNCCa, the non-classicality region is below the corresponding curves.

Finally, we analyze the performance of the above discussed GNCCa when they are applied to the noisy twin beams with unbalanced noise. We assume that the noise is present only in the signal field (*B*_S_ ≠ 0, *B*_i_ = 0). Such twin beams have been theoretically investigated in ref.^39^ with the conclusion that only the twin beams with *B*_S_ < 1 and arbitrary *B*_P_ exhibit non-classicality. The GNCCa ${E}_{{k}_{1},{k}_{2}}^{W}$ and ${E}_{{k}_{1},{k}_{2}}^{p}$ reveal non-classicality only for some noisy twin beams, especially those with smaller amount of the noise [see Fig. 3]. The GNCCa ${E}_{{k}_{1},{k}_{2}}^{W}$ and ${E}_{{k}_{1},{k}_{2}}^{p}$ with *k*_1_ > *k*_2_ are more sensitive to non-classicality as they include higher-order signal-field intensity moments and the elements of the photon-number distribution for greater signal photon numbers, respectively. This is the consequence of the noise present in the signal field. Contrary to this, the GNCCa *M*^*W*^ and *M*^*p*^ are able to indicate non-classicality for all noisy twin beams, as documented in Figs. 3(a) and (b). This means that the GNCCa *M*^*W*^ and *M*^*P*^ allow to reveal non-classicality of all analyzed twin beams.

### Identification of non-classicality of two-mode states beyond a beam splitter

In this section, we address two-mode states that occur at the output ports of a beam splitter with transmissivity *T* assuming an input noisy twin beam. We note that in the boundary cases *T* = 0 and *T* = 1 an input noisy twin beam is just transformed to the beam-splitter output without any modification. On the other hand, the balanced beam splitter with *T* = 1/2 is optimal for the generation of squeezed light in both output ports^28–30^. In general, an arbitrary beam splitter has the potential to generate states that may exhibit both local non-classicality and entanglement.

**Figure 4:**
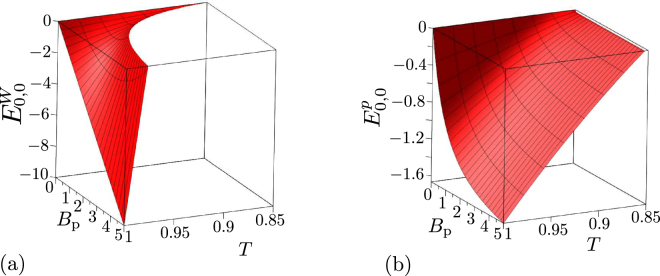
(**a**) GNCC ${E}_{\mathrm{0,0}}^{W}$ and (**b**) GNCC ${E}_{\mathrm{0,0}}^{p}$ as functions of photon-pair number *B*_P_ and beam-splitter transmissivity *T* for noiseless two-mode states beyond the beam splitter.

**Figure 5:**
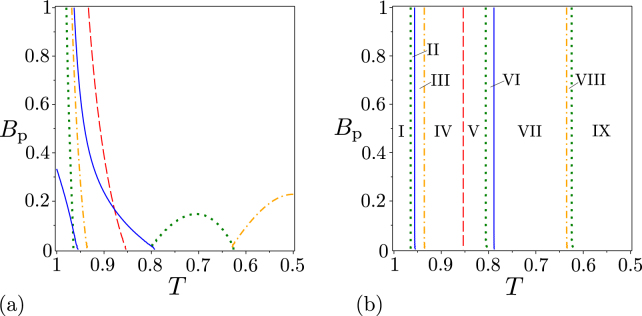
Non-classicality phase diagrams for noiseless two-mode states beyond the beam splitter: (**a**) GNCCa ${E}_{\mathrm{0,0}}^{W}$ (red dashed curve), ${E}_{\mathrm{1,1}}^{W}$ (orange dash-dotted curve), ${E}_{\mathrm{2,2}}^{W}$ (green dotted curve), ${E}_{\mathrm{2,0}}^{W}$ (blue solid curve), and ${M}^{W}$ (red dashed curve) [non-classicality regions are found below the corresponding curves, except that for the GNCC ${E}_{\mathrm{2,0}}^{W}$ occurring between the blue curves] and (**b**) ${E}_{\mathrm{0,0}}^{p}$ (red dashed line), ${E}_{\mathrm{1,1}}^{p}$ (orange dash-dotted lines), ${E}_{\mathrm{2,2}}^{p}$ (green dotted lines), ${E}_{\mathrm{2,0}}^{p}$ (blue solid lines), and *M*^*p*^ (red dashed line) [non-classicality regions for different GNCCa are identified in Table 1] in space (*B*_P_ , *T*).

Similarly as in the previous section, we analyze the noiseless states first. It is interesting that the GNCCa ${E}_{{k}_{1},{k}_{2}}^{p}$ and *M*^*P*^ involving the elements of photon-number distributions factorize as functions of transmissivity *T* and photon-pair number *B*_P_, contrary to the GNCCa ${E}_{{k}_{1},{k}_{2}}^{W}$ and *M*^*W*^ based on intensity moments (see the graphs for ${E}_{\mathrm{0,0}}^{W}$ and ${E}_{\mathrm{0,0}}^{p}$ in Fig. 4 and also the non-classicality phase diagrams in Fig. 5).

In particular, the following formulas can be derived:

32 $$\begin{array}{rcl}{E}_{\mathrm{0,0}}^{p} & = & -\,\mathrm{(1}-8TR)\frac{2{B}_{{\rm{p}}}}{({B}_{{\rm{p}}}+\mathrm{1)}},\\ {E}_{\mathrm{1,1}}^{p} & = & -\,\mathrm{(72}{T}^{2}{R}^{2}-21TR+\mathrm{1)}\frac{8{B}_{{\rm{p}}}^{2}}{{({B}_{{\rm{p}}}+\mathrm{1)}}^{2}},\\ {E}_{\mathrm{2,2}}^{p} & = & -\,\mathrm{(800}{T}^{3}{R}^{3}+340{T}^{2}{R}^{2}-40TR+\mathrm{1)}\frac{72{B}_{{\rm{p}}}^{3}}{{({B}_{{\rm{p}}}+\mathrm{1)}}^{3}}\mathrm{.}\\ {E}_{\mathrm{0,2}}^{p} & = & \mathrm{(144}{T}^{2}{R}^{2}-30TR+\mathrm{1)}\frac{4{B}_{{\rm{p}}}^{2}}{{({B}_{{\rm{p}}}+\mathrm{1)}}^{2}},\end{array}$$

33 $${M}^{p}=-\,\mathrm{(1}-8TR)\frac{{B}_{{\rm{p}}}^{2}}{{({B}_{{\rm{p}}}+\mathrm{1)}}^{2}}\mathrm{.}$$

Due to the factorization, the corresponding non-classicality regions do not depend on photon-pair number *B*_P_. Detailed analysis of the non-classicality regions whose results are summarized in Table 1 has shown that the GNCCa ${E}_{\mathrm{0,0}}^{p}$, ${E}_{\mathrm{1,1}}^{p}$, ${E}_{\mathrm{2,2}}^{p}$, and ${E}_{\mathrm{0,2}}^{p}$ considered together allow to reveal non-classicality of an arbitrary noiseless two-mode state beyond the beam splitter with transmissivity *T∈* [1/2, 1] (and also *T∈* [0, 1/2] due to the symmetry reasons). As the phase diagram plotted in Fig. 5(a) documents, the set of GNCCa ${E}_{\mathrm{0,0}}^{W}$, ${E}_{\mathrm{1,1}}^{W}$, ${E}_{\mathrm{2,2}}^{W}$, and ${E}_{\mathrm{0,2}}^{W}$ involving intensity moments allows to detect non-classicality of all considered states only for small photon-pair numbers *B*_P_. As evident from Figs 4(a) and 5(a), these GNCCa lose their ability to reveal non-classicality with the increasing photon-pair number *B*_P_ .

**Table 1:** Non-classicality regions of GNCCa ${E}_{\mathrm{0,0}}^{p}$, ${E}_{\mathrm{1,1}}^{p}$, ${E}_{\mathrm{2,2}}^{p}$, and ${E}_{\mathrm{0,2}}^{p}$ defined on the beam-splitter transmissivity axis *T* for noiseless two-mode states beyond the beam splitter.

GNCC	Non-classicality region(s)	Corresponding areas in Fig. 5(b)
${E}_{\mathrm{0,0}}^{p}$	$T\in \mathrm{([1}+\mathrm{1/}\sqrt{2}\mathrm{]/2,1]}$	I, II, III, IV
${E}_{\mathrm{1,1}}^{p}$	$T\in \mathrm{[1/2,[1}+\sqrt{15-3\sqrt{17}}\mathrm{/6]/2)}\cup \mathrm{([1}+\sqrt{15+3\sqrt{17}}\mathrm{/6]/2,1]}$	I, II, III, VIII, IX
${E}_{\mathrm{2,2}}^{p}$	$T\in (\approx \mathrm{0.624,}\approx \,\mathrm{0.806)}\cup (\approx \,\mathrm{0.965,1]}$	I, VI, VII, VIII
${E}_{\mathrm{0,2}}^{p}$	$T\in \mathrm{([1}+\mathrm{1/}\sqrt{3}\mathrm{]/2,[1}+\sqrt{30}\mathrm{/6]/2)}$	III, IV, V, VI

The GNCCa ${E}_{\mathrm{0,0}}^{p}$, ${E}_{\mathrm{2,2}}^{p}$ and ${E}_{\mathrm{0,2}}^{p}$ shown in the phase diagram in Fig. 5(b) detect entanglement^15^ and so they lose their ability to reveal global non-classicality as *T* approaches 1/2. The reason is that the entanglement of the considered states is becoming weaker as *T* goes to 1/2 and the state is separable for *T* = 1/2. On the other hand, the GNCC ${E}_{\mathrm{1,1}}^{p}$ safely indicates global non-classicality in the region around $T=\mathrm{1/2}$. This is understood by the fact that the GNCC ${E}_{\mathrm{1,1}}^{p}$ is able to reveal also local non-classicality [${E}_{\mathrm{1,1}}^{p}$ given in Eq. (9) and ${R}_{\mathrm{1,1}}^{p}$ defined in Eq. (12) coincide for separable symmetric (1↔2) states]. We note that the vanishing entanglement in the vicinity of *T* = 1/2 can only be identified by the GNCCa ${E}_{2k\mathrm{,2}k}^{p}$, *k* = 1, 2, .... The greater the number *k* is the two-mode entangled states generated with *T* closer to 1/2 can be revealed. However, this requires the determination of photon-number distributions for greater photon numbers^41^.

**Figure 6:**
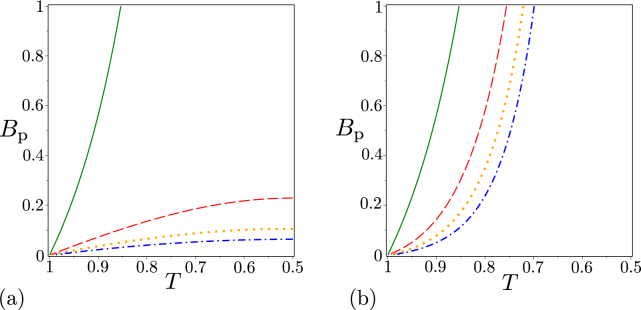
Non-classicality phase diagrams for noiseless two-mode states beyond the beam splitter: (**a**) ${R}_{\mathrm{2,2}}^{W}$ (red dashed curve), ${R}_{\mathrm{4,4}}^{W}$ (orange dotted curve) and ${R}_{\mathrm{6,6}}^{W}$ (blue dash-dotted curve) and (**b**) ${R}_{\mathrm{2,2}}^{p}$ (red dashed curve), ${R}_{\mathrm{4,4}}^{p}$ (orange dotted curve) and ${R}_{\mathrm{6,6}}^{p}$ (blue dash-dotted curve) in space (*B*_P_ , *T*). For comparison, phase diagram of local non-classicality quantifier ${I}_{{\rm{n}}{\rm{c}}{\rm{l}}}^{\mathrm{(1)}}={I}_{{\rm{n}}{\rm{c}}{\rm{l}}}^{\mathrm{(2)}}$ is plotted by green solid curve. Non-classicality regions extend from line *B*_P_ = 0 up to the corresponding curves drawn in the diagrams.

The striking feature of two-mode states beyond the beam splitter is the ability to exhibit local non-classicality. This originates in the bunching effect of photons in a photon pair at a beam splitter. Ideally, two non-distinguishable photons impinging on a balanced beam splitter leave the beam splitter at the same output port. Thus, the original twin beam partly loses its entanglement as it propagates through the beam splitter, but its constituting parts can gain their local non-classicalitites, as quantified by relation (26) for the global non-classicality invariant *I*_ncl_. As local non-classicality arises from pairing of photons, only the LNCCa ${R}_{2k\mathrm{,2}k}^{W}$ and ${R}_{2k\mathrm{,2}k}^{p}$, *k* = 1, 2, ..., allow for detecting local non-classicality. The phase diagram for the local non-classicality quantifier ${I}_{{\rm{nlc}}}^{\mathrm{(1)}}$ in Fig. 6 shows that the majority of the considered states with smaller photon-pair numbers *B*_P_ exhibit local non-classicality. However, the analyzed LNCCa ${R}_{2k\mathrm{,2}k}^{W}$ and ${R}_{2k\mathrm{,2}k}^{p}$ for *k* = 1, 2 and 3, whose phase diagrams are also included in Fig. 6, identify local non-classicality only in some states. As the identifiable states are in the area around *T* = 1/2 they are apparently endowed with stronger local non-classicality. As documented in Fig. 6, the LNCCa ${R}_{2k\mathrm{,2}k}^{W}$, *k* = 1, 2, ..., based on intensity moments are applicable only to weak two-mode fields and they lose their power with the increasing index *k*. Also the LNCCa ${R}_{2k\mathrm{,2}k}^{p}$, *k* = 1, 2, ..., determined from the elements of photon-number distribution gradually lose their power with the increasing index *k*, but they are suitable for indicating local non-classicality in more intense two-mode fields [see Fig. 6(b)]. The LNCC ${R}_{\mathrm{2,2}}^{p}$ is the most powerful among the studied LNCCa and, assuming the beam splitter with fixed transmissivity *T*, it allows to reveal the local non-classicality of two-mode states with photon-pair numbers *B*_P_ lower than

34 $${B}_{{\rm{p}}}=\frac{4TR-1+\sqrt{4TR[4TR-\mathrm{(7}+\sqrt{33})]+1}}{\mathrm{2(2}T-{\mathrm{1)}}^{2}}\mathrm{.}$$

**Figure 7:**
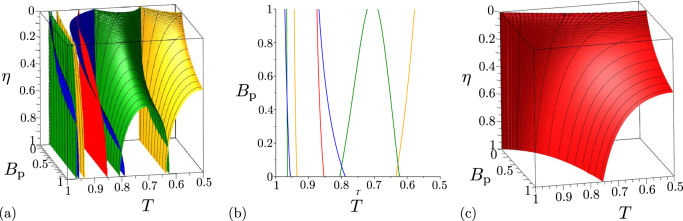
Non-classicality phase diagrams for noiseless two-mode states beyond the beam splitter: (**a**) GNCCa ${E}_{\mathrm{0,0}}^{p}$ (red surface), ${E}_{\mathrm{1,1}}^{p}$ (orange contour surface), ${E}_{\mathrm{2,2}}^{p}$ (green contour surface), ${E}_{\mathrm{2,0}}^{p}$ (blue contour surface), and ${M}^{p}$ (red surface), (**b**) plane *η* = 0.5 in the phase diagrams plotted in (**a**) and (**c**) LNCC ${R}_{\mathrm{2,2}}^{p}$ in space (${B}_{{\rm{p}}}$, $T$, $\eta$). We note that the planes *η* = 0 and *η* = 1 in the phase diagrams in (**a**) [(**c**)] are plotted in Figs. 5(a) and (b) [Figs. 6(a) and (b) by red dashed curves], respectively. Non-classicality regions occur below the corresponding surfaces, only that for the GNCC ${E}_{\mathrm{2,0}}^{p}$ in (**a**) is found between blue contour surfaces.

In real experimental identification of both global and local non-classicalities finite detection efficiencies are an important issue. The GNCCa as well as LNCCa based on intensity moments are not sensitive to detection efficiency *η* because the moments in these criteria are only synchronously rescaled with appropriate powers of efficiency *η*. Contrary to this, the GNCCa and LNCCa containing the elements of photon-number distribution suffer from the finite detection efficiency *η*. Gradual loss of the power to resolve nonclassical states with decreasing detection efficiency *η* is documented in Fig. 7(a) for the GNCCa ${E}_{\mathrm{0,0}}^{p}$, ${E}_{\mathrm{1,1}}^{p}$, ${E}_{\mathrm{2,2}}^{p}$, ${E}_{\mathrm{2,0}}^{p}$, and *M*_*P*_ and in Fig. 7(c) for the LNCC ${R}_{\mathrm{2,2}}^{p}$. Except for the GNCC ${E}_{\mathrm{2,0}}^{p}$, the set of nonclassical two-mode states identified by the other analyzed GNCCa and LNCCa only diminishes with decreasing detection efficiency *η*. For the GNCC ${E}_{\mathrm{2,0}}^{p}$, nonclassical two-mode states with decreasing photon-pair numbers *B*_P_ are gradually identified as the detection efficiency *η* decreases [compare the corresponding phase diagrams in Figs. 5(a) and (b)].

In the limit *η* → 0, the phase diagrams of the GNCCa and LNCCa based on the elements of photon-number distribution coincide with those written for intensity moments. This property has its origin in the form of the Mandel photodetection formula that provides the following relation for small detection efficiencies *η*:

35 $${n}_{1}!{n}_{2}!p({n}_{1},{n}_{2})\approx {\eta }^{{n}_{1}+{n}_{2}}\langle {W}_{1}^{{n}_{1}}{W}_{2}^{{n}_{2}}\rangle \mathrm{.}$$

The process of gradual loss of the ability to detect non-classicality with decreasing detection efficiency *η* can be treated even analytically for individual GNCCa and LNCCa. For example, we have for the GNCCa ${E}_{\mathrm{0,0}}^{p}$ and ${E}_{\mathrm{0,0}}^{W}$

36 $${E}_{\mathrm{0,0}}^{p}=K[{E}_{\mathrm{0,0}}^{W}-2\eta \mathrm{(2}-\eta ){B}_{{\rm{p}}}^{2}]$$

and $K={\eta }^{2}\mathrm{/[1}+\eta \mathrm{(2}-\eta ){B}_{{\rm{p}}}{]}^{2}$ is a positive constant. We have ${E}_{\mathrm{0,0}}^{p}={\eta }^{2}{E}_{\mathrm{0,0}}^{W}$ for *η* approaching 0.

**Figure 8:**
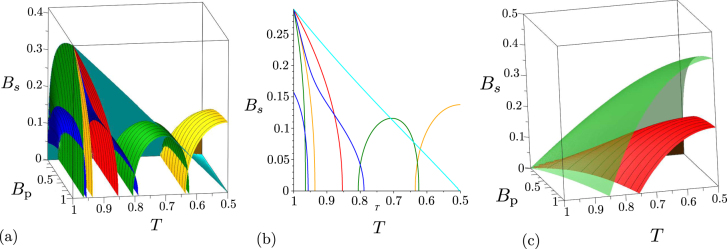
Non-classicality phase diagrams for two-mode states beyond the beam splitter with input noisy twin beams with balanced noise *B*_S_ = *B*_i_: (**a**) GNCCa ${E}_{\mathrm{0,0}}^{p}$ (red contour surface), ${E}_{\mathrm{1,1}}^{p}$ (orange contour surface), ${E}_{\mathrm{2,2}}^{p}$ (green contour surface), ${E}_{\mathrm{2,0}}^{p}$ (blue contour surface), *M*^*P*^ (red contour surface) and entanglement quantifier *I*_ent_ (cyan surface), (**b**) plane *B*_P_ = 0.2 in the phase diagrams plotted in (**a**) and (**c**) LNCC ${R}_{\mathrm{2,2}}^{p}$ (red contour surface) and local non-classicality quantifiers ${I}_{{\rm{n}}cl}^{\mathrm{(1)}}={I}_{{\rm{n}}cl}^{\mathrm{(2)}}$ (green surface) in space (${B}_{{\rm{p}}}$, $T$, ${B}_{{\rm{s}}}$). Non-classicality regions occur in (**a**) and (**c**) below the corresponding surfaces, except for the GNCC ${E}_{\mathrm{2,0}}^{p}$ in (**a**) for which the non-classicality region is surrounded by blue contour surfaces.

Two-mode states occurring beyond the beam splitter with an impinging noisy twin beam can be both locally nonclassical and entangled. However, the numbers *B*_S_ = *B*_i_ of noise photons cannot exceed the value $\sqrt{{B}_{{\rm{p}}}({B}_{{\rm{p}}}+\mathrm{1)}}-{B}_{{\rm{p}}}$ for twin beams with balanced noise. It holds that the entangled two-mode states are generated in the areas around *T* = 1 and *T* = 0 when the input noise cannot be neglected. With the increasing numbers *B*_S_ = *B*_i_ of noise photons the areas containing entangled states shrink towards the points *T* = 1 and *T* = 0 [see Fig. 8(a)]. On the other hand, two-mode states exhibiting local non-classicality occur in the area around *T* = 1/2. With the increasing numbers *B*_S_ = *B*_i_ of noise photons this area diminishes [see Fig. 8(c)].

Neither the entangled two-mode states nor the locally-nonclassical two-mode states can be completely identified by the analyzed GNCCa and LNCCa. For example, the LNCC ${R}_{\mathrm{2,2}}^{p}$, that performs the best, identifies local non-classicality only for two-mode states with the numbers *B*_S_ = *B*_i_ of noise photons below the value

37 $${B}_{{\rm{s}}}={B}_{{\rm{i}}}=\frac{1}{2}[\sqrt{16TR{B}_{{\rm{p}}}({B}_{{\rm{p}}}+1)+4\sqrt{\sqrt{33}-5}\sqrt{TR{B}_{{\rm{p}}}({B}_{{\rm{p}}}+1)}+1}-2{B}_{{\rm{p}}}-1].$$

**Figure 9:**
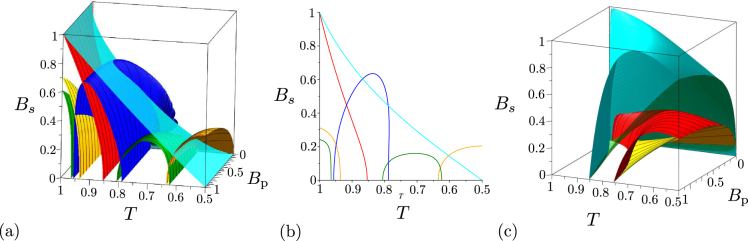
Non-classicality phase diagrams for two-mode states beyond the beam splitter with input noisy twin beams with unbalanced noise: (**a**) GNCCa ${E}_{\mathrm{1,1}}^{p}$ (orange contour surface), ${E}_{\mathrm{2,2}}^{p}$ (green contour surface), ${E}_{\mathrm{0,2}}^{p}$ (blue contour surface), *M*^*P*^ (red contour surface), and entanglement quantifier *I*_ent_ (cyan surface), (**b**) plane *B*_P_ = 0.2 in the phase diagrams plotted in (**a**) and (**c**) LNCCa ${R}_{\mathrm{2,2}}^{{p}_{1}}$ (orange contour surface), ${R}_{\mathrm{2,2}}^{{p}_{2}}$ (red contour surface) and local non-classicality quantifiers ${I}_{{\rm{n}}{\rm{c}}{\rm{l}}}^{\mathrm{(1)}}$ (green surface) and ${I}_{{\rm{n}}{\rm{c}}{\rm{l}}}^{\mathrm{(2)}}$ (cyan surface) in space (*B*_p_,*T*,*B*_s_). Non-classicality regions in (**a**) and (**c**) occur below the corresponding surfaces.

When the noisy twin beams with unbalanced noise (*B*_S_ ≠ 0, *B*_i_ = 0) are assumed at the beam splitter, the generated two-mode states behave similarly as those analyzed in the case of twin beams with balanced noise. To observe non-classicality, the input noisy twin beams cannot contain more than one noise signal photon on average (*B*_S_ < 1). Contrary to the case with balanced noise, only the GNCC *M*^*P*^ is powerful in identifying entanglement for two-mode states beyond the beam splitter with transmissivity *T* close to 1 and 0 in this case [see phase diagrams in Fig. 9(a)]. Also the GNCCa ${E}_{\mathrm{0,2}}^{p}$ and ${E}_{\mathrm{2,0}}^{p}$ perform differently. For two-mode states generated for $T\,\in \,\mathrm{(1/2,}\,\mathrm{1]}$ the GNCC ${E}_{\mathrm{0,2}}^{p}$ is more efficient and it allows to reveal the entanglement of all states detectable by the GNCC ${E}_{\mathrm{2,0}}^{p}$. Also the best performing LNCCa ${R}_{\mathrm{2,2}}^{{p}_{1}}$ and ${R}_{\mathrm{2,2}}^{{p}_{2}}$, that reveal local non-classicality, give different results in different areas of their phase diagrams [see Fig. 9(c)]. Whereas the LNCC ${R}_{\mathrm{2,2}}^{{p}_{1}}$ is more suitable for two-mode states generated for *T* ∈ (0, 1/2], the LNCC ${R}_{\mathrm{2,2}}^{{p}_{2}}$ is more powerful for indicating local non-classicality of two-mode states reached for *T* ∈ (1/2, 1], as documented in Fig. 9(c). We note that the mean number *B*_S_ of noise signal photons of the input twin beam is divided into the output beam-splitter ports such that *TB*_S_ mean noise photons leave mode 1 and *RB*_S_ mean noise photons occur in mode 2.

## Conclusions

We have analyzed the performance of several local and global non-classicality criteria written for intensity moments and elements of photon-number distributions and applied to noisy twin beams and other two-mode states derived from noisy twin beams by using a beam splitter. It has been shown that the non-classicality criteria based on the elements of photon-number distributions exhibit in general better performance in revealing both local and global non-classicalities compared to those containing intensity moments. However, these criteria lose their power with decreasing detection efficiencies and they give the same results as the criteria based on intensity moments for low detection efficiencies. Both types of criteria contain one criterion that reveals the entanglement as one of the forms of global non-classicality for all entangled noisy twin beams. Contrary to this, not all locally and globally nonclassical two-mode states occurring beyond the beam splitter are detectable by the analyzed non-classicality criteria. However, simultaneous application of several criteria gives a good chance for revealing possible non-classicality of an unknown two-mode state generated beyond the beam splitter.
