# Publisher Correction: Experimental identification of non-classicality of noisy twin beams and other related two-mode states

**DOI:** 10.1038/s41598-018-26339-y

**Published:** 2018-05-31

**Authors:** Ievgen I. Arkhipov, Jan Peřina

**Affiliations:** RCPTM, Joint Laboratory of Optics of Palacký University and Institute of Physics of CAS, Faculty of Science, Palacký University, 17.listopadu 12, 771 46 Olomouc, Czech Republic

Correction to: *Scientific Reports* 10.1038/s41598-018-19634-1, published online 23 January 2018

The previous version of this Article contained errors in the Introduction under the subheading ‘Twin beam and its transformation on a beam splitter’,

$${\boldsymbol{\lambda }}\,={(1,{{\rm{\lambda }}}_{1},{{\rm{\lambda }}}_{2},{{\rm{\lambda }}}_{1}{{\rm{\lambda }}}_{2})}^{{\rm{T}}}$$

now reads:

$${\boldsymbol{\lambda }}\,\equiv {(1,{{\rm{\lambda }}}_{1},{{\rm{\lambda }}}_{2},{{\rm{\lambda }}}_{1}{{\rm{\lambda }}}_{2})}^{{\rm{T}}}$$

Additionally, the Publisher Correction published on 30^th^ April 2018 contains errors.

“This Article contains errors in Equation 18.

$${{\rm{G}}}_{{\mathscr{N}}}({{\rm{\lambda }}}_{1},{{\rm{\lambda }}}_{1})=\frac{1}{{[{{\rm{\lambda }}}^{{\rm{T}}}{\bf{K}}{\rm{\lambda }}]}^{1/2}}$$

and $${\rm{\lambda }}\,\equiv {(1,{{\rm{\lambda }}}_{1},{{\rm{\lambda }}}_{2},{{\rm{\lambda }}}_{1},{{\rm{\lambda }}}_{2},)}^{{\rm{T}}}$$.

should read:

$${{\rm{G}}}_{{\mathscr{N}}}({{\rm{\lambda }}}_{1},{{\rm{\lambda }}}_{1})=\frac{1}{{{[{\rm{\lambda }}}^{{\rm{T}}}{\bf{K}}{\rm{\lambda }}]}^{1/2}}$$

and $${\rm{\lambda }}\,\equiv {(1,{{\rm{\lambda }}}_{1},{{\rm{\lambda }}}_{2},{{\rm{\lambda }}}_{1}{{\rm{\lambda }}}_{2})}^{{\rm{T}}}$$.”

should read:

“The original version of this Article contained errors in Equation 18.

$${{\rm{G}}}_{{\mathscr{N}}}({{\rm{\lambda }}}_{1},{{\rm{\lambda }}}_{2})=\frac{1}{{[{{\boldsymbol{\lambda }}}^{{\rm{T}}}{\bf{K}}{\boldsymbol{\lambda }}]}^{1/2}}$$

and $${\boldsymbol{\lambda }}\,={(1,{{\rm{\lambda }}}_{1},{{\rm{\lambda }}}_{2},{{\rm{\lambda }}}_{1},{{\rm{\lambda }}}_{2},)}^{{\rm{T}}}$$.

now reads:

$${{\rm{G}}}_{{\mathscr{N}}}({\lambda }_{1},{\lambda }_{2})=\frac{1}{{[{{\boldsymbol{\lambda }}}^{{\rm{T}}}{\bf{K}}{\boldsymbol{\lambda }}]}^{1/2}}$$

and $${\boldsymbol{\lambda }}\,\equiv {(1,{{\rm{\lambda }}}_{1},{{\rm{\lambda }}}_{2},{{\rm{\lambda }}}_{1}{{\rm{\lambda }}}_{2})}^{{\rm{T}}}$$.”

